# Editorial: Diagnosis and treatment for arteriosclerosis and thrombosis

**DOI:** 10.3389/fsurg.2025.1619947

**Published:** 2025-05-23

**Authors:** Yang Liu, Yao Liu, Yihao Zheng

**Affiliations:** ^1^ Global Institute of Future Technology, Shanghai Jiao Tong University, Shanghai, China; ^2^ School of Mechanical Engineering, North University of China, Taiyuan, China; ^3^ Mechanical and Materials Engineering, Worcester Polytechnic Institute, Worcester, MA, United States

**Keywords:** peripheral artery disease (PAD), chronic limb-threatening ischemia (CLTI), spinal cord stimulation, vessel preparation, hybrid approach

Editorial on the Research Topic Diagnosis and treatment for arteriosclerosis and thrombosis

Arteriosclerosis and thrombosis are leading contributors to morbidity with peripheral artery disease (PAD) affecting over 110 million individuals worldwide (1). Despite recent advances in endovascular approaches, challenges persist in managing complex lesions such as those classified as the Trans-Atlantic Inter-Society Consensus (TASC) II type C/D. Determining the optimal treatment strategy to achieve a high long-term patency rate while minimizing short-term risks remains an area of active investigation and discussion (2). This editorial summarizes the findings of three studies published in this Research Topic, which highlight innovations in vessel preparation, hybrid approaches to complex lesions, and spinal cord stimulation (SCS) for PAD with chronic limb-threatening ischemia (CLTI).

Vessel preparation has almost become an essential step prior to stenting to improve outcomes in endovascular management of PAD, especially for long or calcified lesions. Vessel preparation is achieved by debulking the plaque using atherectomy or modifying the plaque using intravascular lithotripsy or balloons (undersized plain balloons, cutting balloons, or scoring balloons). When using balloons for vessel preparation, suboptimal/uneven expansion in calcified lesions exerting uneven forces is a persisting challenge and calls for the development of new technologies. In a single-arm study of 43 patients, Zhang et al. evaluated the safety and efficacy of using nitinol-constrained chocolate balloons for vessel preparation followed by drug-coated balloons (DCBs) in femoropopliteal artery lesions Zhang et al.. The “pillow effect” of the chocolate balloon increased the balloon-vessel contact area, and distributed the radial load more evenly than plain balloons. This mechanism was validated by the low rate of severe dissection (10.6%) and stent bailout rate (4.3%). The 12-month freedom from clinically driven target lesion revascularization rate was 78.7%, similar to the rate (78.8%) reported in the Chocolate Touch study (3). This study provided initial clinical data supporting the two-step strategy of combining chocolate balloons and DCBs.

For complex lesions classified as TASC II type C/D, endovascular approaches have suffered from technical failures due to challenges such as lesion crossing, the need for multiple devices for long lesions, and heavy calcifications. Some patients with TASC II type C/D lesions are poor candidates for extensive open surgery due to comorbidities. The hybrid approach combines the durability of open surgery for critical segments with the minimal invasiveness of endovascular approaches for others. However, quantifiable mechanistic explanations, especially regarding how open surgery changes lesion complexity intraoperatively, have rarely been reported. In a cohort of 103 patients, Park et al. demonstrated that femoral endarterectomy (FE) resulted in a reduction of TASC II type from C/D to A/B in 91% of the cases Park et al.. The one- and five-year primary patency rates were 89% and 77%, respectively, demonstrating great efficacy. This study reinforced the use of femoral endarterectomy and iliac angioplasty (FEIA) as a viable option for managing multilevel lesions and first reported the “downgrading” effect of FE. In a recent retrospective study including 26 patients with aorto-iliac occlusive disease with TASC II type C/D lesions, the pure endovascular approaches also demonstrated great efficacy, with one- and five-year primary patency rates of 100% and 91%, respectively (4). These exceptionally high patency rates were likely due to the lack of femoral lesion involvement, and implied that the efficacy of the hybrid approach depends on the specific pattern of PAD. While endovascular technologies have dramatically improved over the years, the hybrid approach retains a critical role in managing complex lesions.

PAD can progress to its most severe form, CLTI. Patients with CLTI are often not eligible for open surgery due to comorbidities, and the efficacy of endovascular approaches is limited as the occlusion is usually diffuse, multi-level, and heavily calcified, presenting significant technical challenges and leading to low intraprocedural success rates (5). These no-option patients lack effective therapeutic solutions and are faced with a one-year amputation rate as high as 30%–45% (6). To relieve pain, clinical evidence supports the use of SCS but its effects on limb salvage have been under debate. In the pioneering ESES trial, SCS in addition to the best medical care did not prevent amputation in patients with CLTI (7). In a small cohort of 13 diabetic patients with CLTI who were ineligible for vascular reconstruction, Cyrek et al. reported a 92.3% one-year limb salvage rate and a 75% ulcer healing rate with SCS implantation Cyrek et al.. The pain score decreased from 7.8 to 3.5 and quality-of-life scores increased by approximately 50%. The non-significant change in the Ankle-Brachial Index during the one-year follow-up period implies that SCS likely did not impact macrovascular flow, and the relieved pain and improved quality of life may be due to the SCS' microcirculatory and pain modulation effects. These results, although preliminary and with a short follow-up period, provide real-world evidence that SCS may provide tangible benefits to no-option patients with CLTI. The study's primary limitation, as acknowledged by the authors, is its design: a retrospective analysis of a very small, single-center cohort without a control group.

In summary, the studies highlighted addressed critical challenges in managing arteriosclerosis and thrombosis and emphasized the importance of tailored approaches with patients with complex PAD and CLTI.
